# A Case Report of Emphysematous Pyelonephritis as a First Presentation of Diabetes Mellitus

**DOI:** 10.5812/ircmj.10384

**Published:** 2013-12-05

**Authors:** Amal Ali Nasr, Ashraf Gaber Kishk, Ehab Makram Sadek, Socrates Mathew Parayil

**Affiliations:** 1 Medical Department, Amiri Hospital, Kuwait City, Kuwait; 2 Histopathology Department, Amiri Hospital, Kuwait City, Kuwait

**Keywords:** Pyelonephritis, Diabetes Mellitus, Nephrectomy

## Abstract

Emphysematous Pyelonephritis (EPN) is an acute suppurative Infection of the kidney. It is an uncommon infection, occurs mostly in patients with diabetes and a predilection for females. It has a high fatality rate; therefore, aggressive medical, early intervention or surgical approach is recommended. We present here a woman with no previous medical history presented with uncontrolled hyperglycemia for the first time associated with EPN.

## 1. Introduction

EPN is a severe necrotizing infection of the kidney and its surroundings. The first case was described in 1898 by Kelly and MacCullum (1). Most of the cases are reported in diabetics, about 90% reported according to different series; obstructive uropathy is the other contributing factor in other cases. It is mostly unilateral but in 10% is bilateral. Patients are usually critically ill, with a high mortality rate ranging from 69% to 18% depending on various elements that would be discussed later (2). Computed tomography (CT) remains the optimal diagnostic radiological investigation. Escherichia coli is the most common causative pathogen isolated on urine or pus culture in nearly 70% of the reported cases (3). Aggressive treatment with broad spectrum antibiotics is recommended. Early interference with nephrectomy was almost a mandatory approach. This trend has changed in recent studies because of advances in interventional radiology and advent of stronger antibiotics, reserving nephrectomy for patients who do not respond to conservative measures. The case presented is a classical scenario of the clinical presentation with more than two risk factors which required early nephrectomy.

## 2. Case Report

A 42-year-old Indian female was admitted with clinical picture of acute pyelonephritis. She had a 7 day history of fever, left loin pain and vomiting. She had no previous significant history. Physical examination revealed a sick patient with high fever and tender left loin. Basic investigations on admission reported high blood sugar (36 mmol/L) with HbA1c of 16.7%. Urine routine and microscopy showed excess WBC but negative results for ketones. She had acute renal failure (s.urea: 11.9 mmol/L, Creatinine 243 umol/L). Arterial blood gas revealed normal pH with normal oxygen saturation. CBC (complete blood count) showed leukocytosis (17 × 10^9^/L) with dominant polymorphs, normal hemoglobin, and thrombocytopenia (92 × 10^9^/L).

She had slightly impaired liver function tests with normal bilirubin, high alkaline phosphatase of 331 IU/L, and normal coagulation profile. Chest X-ray showed minimal left pleural effusion. Plain KUB revealed no significant abnormality. Urgent ultrasound reported normal right kidney but left kidney was not visualized because of a lot of gases.

She was diagnosed as acute pyelonephritis with sepsis and uncontrolled hyperglycemia. She was started empirically on cefotaxime and amikacin injection, placed on intravenous fluids and insulin infusion pump. After 24 hours blood and urine cultures reported growth of resistant E coli (ESBL positive). Antibiotics were changed to meropenem injection.

Despite full supportive measures, she continued to be ill, highly febrile, with further deterioration of renal functions, and persistent leukocytosis with thrombocytopenia.

**Figure 1.**
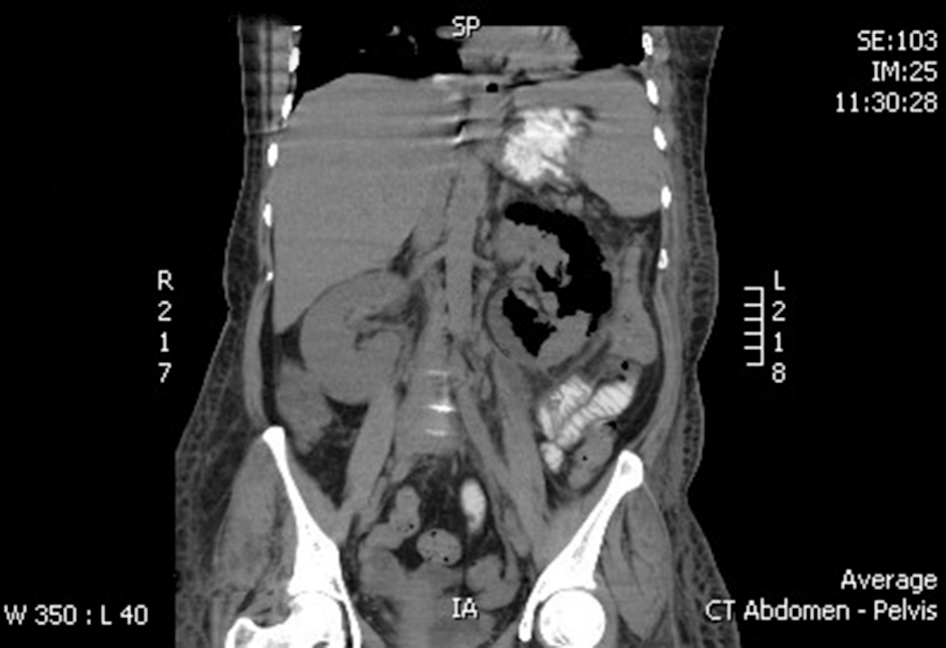
CT Features of Left Sided Emphysematous Pyelonephritis (Coronal Cut)

**Figure 2.**
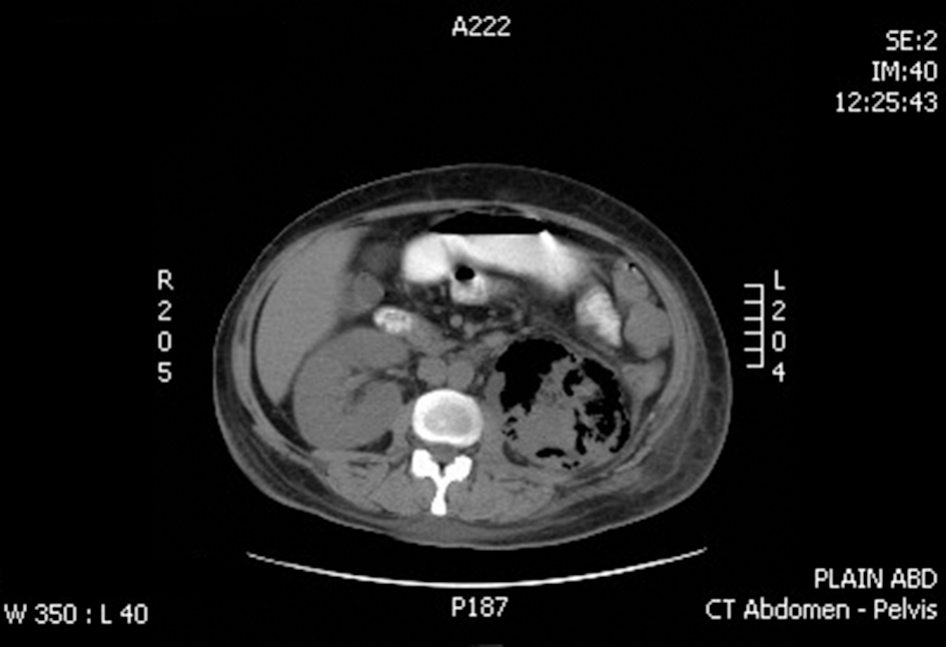
CT Features of Left Sided Emphysematous Pyelonephritis (Axial Cut)

Plain CT-abdomen was performed after 72 hours. It reported mild left pleural effusion with enlarged left kidney heterogeneous with multiple areas of air density and thickened Gerotas fascia with perinephric fat stranding. A picture of left–sided emphysematous nephritis. The right kidney had normal findings (Figures 1 and 2). 

**Figure 3.**
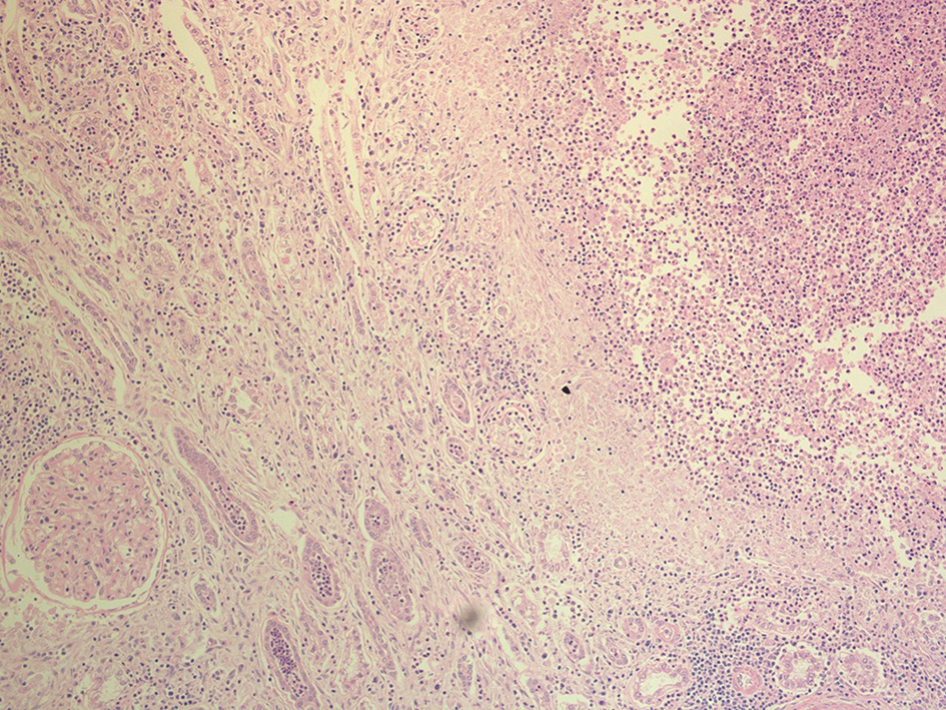
The Histopathology of the Removed Kidney Showed Features of Acute Pyelonephritis With Extensive Patchy Areas of Suppurative Inflammation and Necrosis With Adjacent Renal Tissue Displaying Intratubular and Interstitial Inflammatory Cells Including Neutrophilic Infiltrates (H&E Stain,10X Magnification)

**Figure 4.**
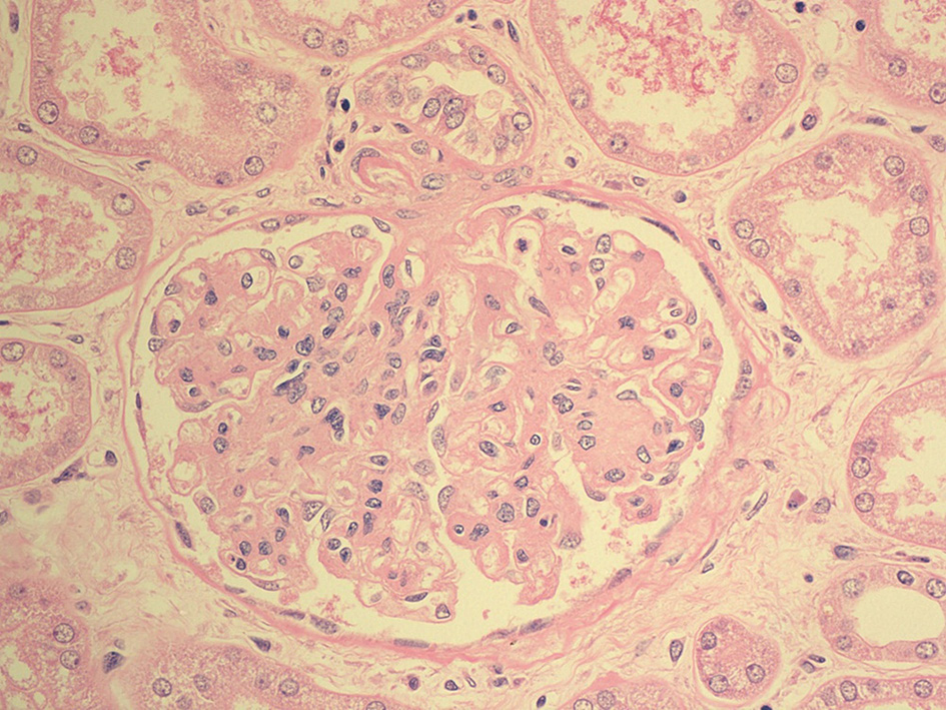
The Histopathology of the Glomeruli Showing Diffuse Increase in Mesangial Matrix With Thickened Capillary Walls and a Nodule (Kimmelstiel- Wilson Lesion) of Diabetic Glomerulosclerosis (H&E stain, 40X Magnification)

Based on the clinical picture and the radiological findings the urology team was involved and she was scheduled for urgent nephrectomy which was performed after 96 hours of admission. She had severely edematous perinephric space. The removed kidney was gangrenous, and a drain was left which was removed after 12 days. The histopathology of the removed kidney showed features of acute pyelonephritis with extensive patchy areas of suppurative inflammation and necrosis (Figure 3), also glomeruli showed diffuse increase in mesangial matrix with thickened capillary walls, and a nodule (Kimmelstiel-Wilson lesion) of diabetic glomerulosclerosis (Figure 4). 

She had an uneventful recovery, required 5 days in ICU, recovering gradually. She received meropenem for 21 days. Renal and liver function tests and CBC normalized. Blood sugar became normal with small dose of metformin. She was discharged with an excellent prognosis.

## 3. Discussion

EPN is a uniformly fatal illness if left untreated. Patients who are treated medically have a higher mortality rate than those treated surgically, 70% vs. 30%. Most cases are associated with uncontrolled diabetes mellitus, around 90% in different series, obstructive uropathy is the other predisposing factor (1, 2). The most common causative organisms are Escherichia coli, Klebsiella pneumoniae, proteus mirabilis, and pseudomonas aeruginosa (3-5). EPN predisposing factors in diabetics include uncontrolled hyperglycemia, presence of glucose- fermenting bacteria, impaired vascular supply with poor kidney perfusion, and impaired immunity. In nondiabetic patients obstruction of the urinary tract is the underlying factor (1, 6). Clinical manifestations are similar to patients presenting as acute pyelonephritis but usually not responding to medical treatment. Confirmation of the diagnosis is by radiologic study. Plain X-ray abdomen can be more specific than sonography in detecting air in the renal collecting system but both have series limitations because of superimposition of gas from the bowel or retroperitonium. CT abdomen is a more specific and sensitive tool, and has been recommended as the most useful diagnostic modality. Radionuclide imaging is the most specific and sensitive modality for assessing differential function when nephrectomy is decided (1, 4, 5). Several patterns have been described as CT findings including streaky, streaky and mottled, and streaky and bubbly. Gas can extend into perinephric area, renal vein or inferior vena cava (5). Two distinct types of EPN had been described. Type 1 characterized by renal parenchymal necrosis with absence of fluid content or presence of a streaky /mottled gas pattern. It has a fulminant course and mortality rate of 18%. Type 2 is characterized by presence of renal or perirenal fluid accompanied by bubbly gas pattern or gas in the collecting system (1, 4, 7). Haung et al. adopted a modified staging system, based on CT findings, as four classes correlated with severity (5).

In a meta-analysis of seven cohort studies, including 175 patients, risk factors for mortality were analyzed. Overall mortality rate was 25 % (range from 11% - 42%).

Risk factors associated with higher mortality were as follows: conservative treatment, bilateral EPN, type one EPN, thrombocytopenia, and systolic blood pressure less than 90 mmHg, serum creatinine greater than 230 umol/L (2.5mg/dL) and disturbed consciousness. There was no association with diabetes mellitus (7). These risk factors should be taken into consideration when deciding on treatment approach.

Treatment should be aggressive, starts with vigorous fluid resuscitation, antibiotic therapy, and control of blood sugar and electrolytes. Before the advent of interventional radiology, early surgery and nephrectomy was a mandatory procedure (1). Medical treatment was reserved for patients when surgery is contraindicated. Recent several reports have published several cases where percutaneous drainage resulted in successful outcome but in carefully selected patients (2, 4, 6, 8, 9). Aggressive early surgical approach remains the gold standard in patients with risk factors.

The case reported here demonstrates multiple risk factors associated with high mortality if medical conservative approach was adopted, including high serum creatinine, thrombocytopenia and type 1 EPN. EPN is a rare infection with a high mortality rate if not approached aggressively. An early suspicion of EPN should be raised when a poor response to antibiotic therapy is noted in a patient with diabetes thought to have uncomplicated pyelonephritis. Early imaging studies should be performed, and surgery pursued early in patients who are at high risk of mortality.
